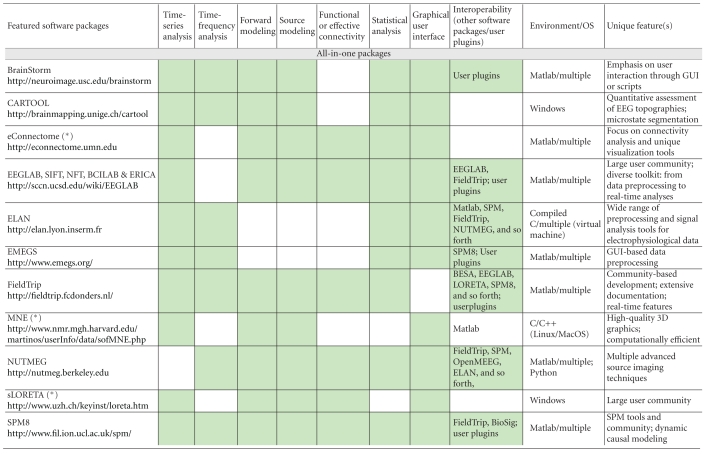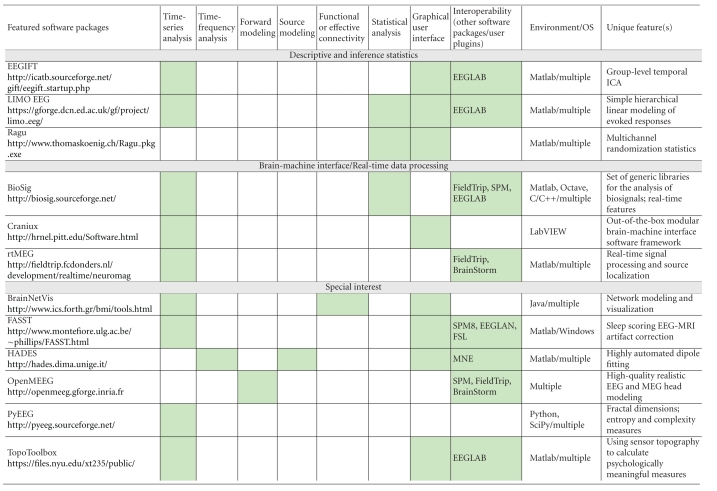# Academic Software Applications for Electromagnetic Brain Mapping Using MEG and EEG

**DOI:** 10.1155/2011/972050

**Published:** 2011-07-13

**Authors:** Sylvain Baillet, Karl Friston, Robert Oostenveld

**Affiliations:** ^1^Departments of Neurology and Biophysics, Medical College of Wisconsin, Milwaukee, WI 53226, USA; ^2^The Wellcome Trust Centre for Neuroimaging, UCL Institute of Neurology, Queen Square, London WC1N 3BG, UK; ^3^Donders Institute for Brain, Cognition and Behaviour, Centre for Cognitive Neuroimaging, Radboud University Nijmegen, 6500 HB Nijmegen, The Netherlands

##  The Hard Work and the Software

In the last decade, the imaging neuroscience community has become very amenable to data sharing and the exchange of people and ideas across imaging modalities. The development of academic software has accompanied this openness and has served to disseminate new and rapidly evolving methodologies and to facilitate the reproducibility of data analyses. The academic recognition of this effort is not straightforward. This special issue features 20 software contributions, all freely available to the research community. It represents a vast swath of MEG and EEG applications and research topics: from real-time data analysis for brain-machine interface applications to all-in-one applications for imaging, modeling and visualization. We hope this special issue offers a technical and citable reference to users of these software packages and will encourage more scientists to share and disseminate their ideas in this pragmatic fashion. Software development in academia has often been equated with prototyping *ad hoc* computing solutions to specific empirical problems. Until recently, in neuroscience, as in other highly specialized scientific fields, computer science skills have been regarded as insufficient to support the distribution of software packages beyond the institutions that developed them. Research groups often considered in-house software literally, as pieces of intellectual property that should not be shared with a community of potential competitors.

The explosion of neuroimaging advances in the 1990s triggered a shift in this perspective. Imaging neuroscience is essentially multidisciplinary: neuroscientists design paradigms, collect and then analyze data; physicists develop and implement new instrumentation, while mathematicians and theorists develop bespoke statistical analyses and the models needed to improve our understanding of how data are generated. In this context, software has become a vector for sharing, cross-fertilizing, disseminatiing and replicating scientific results. In essence, this new regard for software has paralleled the interest in open-source developments (Linux, Python libraries, etc.), public licensing (e.g., GNU General Public License, BSD) in computer science, and the emerging model of open-access peer-reviewed scientific publishing. It also has benefited enormously from rapid and accessible prototyping environments such as Matlab, which have enabled many neuroscientists to write robust and sophisticated software. We note that although these environments may not be free, their distributors usually grant privileged pricing options to academia. We are also aware that entirely free alternatives exist and are developing constantly (e.g., Octave, SciPy.org, and other Python initiatives). 

Commercial software packages are readily available for some imaging modalities and could represent the best choice (or the only alternative) with respect to support, documentation, quality assurance, performance, and most importantly, regulatory and clinical compliance. However, in a field where methodological developments are evolving constantly to address new imaging challenges and techniques, the flexibility and reactivity of academic software ensure that it remains at the forefront of advances in brain imaging. 

Nevertheless, software development in academia comes with a price. It takes a considerable amount of human resources and skills, which are sometimes not fully appreciated by scientific peers and funding agencies. Although the vast majority of investigators have come to realize that good software underwrites the quality of scientific contributions and the profile of institutions disseminating software, the long-term investment of scientists who develop software is difficult to quantify and appreciate. 

We have assembled this special issue to promote these efforts and to offer a bibliographic landmark to their authors. We have restricted the scope of this issue to software for electroencephalography (EEG) and magnetoencephalography (MEG) data analysis, for which a great variety of techniques have been developed. They embrace a scope that is representative of other neuroimaging modalities: from time series analysis to source modeling and image reconstruction; from mapping and localization to the modeling and quantification of functional and effective connectivity; from real-time data analyses to sophisticated group-level statistical inference.

We are grateful to the authors of the 20 manuscripts presented herewith (Table 1) and to our anonymous reviewers. We have organized the contents of this issue according to the nature of the software featured: all-in-one packages, tools for descriptive and inference statistics, tools for brain-machine interface applications and real-time data processing, and special-interest tools that usually feature a unique application. We believe these contributions offer a balanced reflection of the open issues and scientific challenges in MEG/EEG research. They also reveal the multiple faces of what academic software has come to offer: from specific toolkits that embody the methodological developments of a particular research group, to integrated software applications embracing multiple features, sophisticated user interfaces and libraries. It is worth mentioning that some of these tools have been developed and supported for over a decade. The requirements for acceptance to this special issue were, beyond technical validity, that the featured software is available for download and is free of charge and that user documentation is readily available. 

We truly hope this special issue celebrates the communal efforts in our field and becomes the academic citation reference for the developers of the featured software packages and their users.

*Sylvain Baillet* *Karl Friston* *Robert Oostenveld*

## Figures and Tables

**Table 1:** Alphabetic list of the software contributions featured in this special issue. Three further prominent software packages (eConnectome, MNE and sLORETA) have been added, for reference and convenience of comparison. The main features of all software packages are itemized in terms of time-series analysis (from basic preprocessing of raw data to dynamic systems metrics), time-frequency analysis and decomposition, MEG/EEG forward modeling and/or source modeling (dipole fitting, beamforming/spatial filters, and source imaging), functional or effective connectivity measures and modeling, statistical analysis and inference, graphical user interface versus scripting libraries, interoperability with other software packages/the possibility for users to develop plugins, the computing environment and the operating system(s) (OS) required, and definitive feature(s) that characterizes each software package. Asterisks are for packages that are not featured as full-length articles in this special issue. Green cells indicate which attributes apply to each package.